# Correlations between NLR, NHR, and clinicopathological characteristics, and prognosis of acute ischemic stroke

**DOI:** 10.1097/MD.0000000000033957

**Published:** 2023-06-16

**Authors:** Feng Zhu, Yan Ji, Jiang-Hua Song, Guo-Xiang Huang, Yun-Feng Zhang

**Affiliations:** a Department of Neurology, Nantong Third People’s Hospital, Affiliated Nantong Hospital 3 of Nantong University, Nantong, Jiangsu Province, China; b Department of Neurology, Affiliated Hospital of Nantong University, Nantong, Jiangsu Province, China.

**Keywords:** acute ischemic stroke, neutrophil/HDL cholesterol ratio, neutrophil/lymphocyte ratio, prognosis

## Abstract

Neuroinflammation plays an essential role in the process of acute ischemic stroke (AIS) injury repair. The current study seeks to investigate the relationship between the neutrophil/lymphocyte ratio (NLR) and neutrophil/high-density lipoprotein cholesterol ratio (NHR) and AIS disease severity and short-term prognosis. As such, the primary aim of this study is to improve AIS diagnosis and treatment. A total of 136 patients with AIS at the Nantong Third People’s Hospital were retrospectively analyzed. The inclusion criteria comprised patients with ischemic stroke admitted to the hospital <24 hours after symptom onset. Baseline, clinical, and laboratory data were collected from all patients within 24 hours of admission. Univariate, multivariate and receiver operating characteristic curve analysis were performed to determine the relationship between NLR, NHR, AIS severity, and short-term prognosis. NLR (odds ratio [OR] = 1.448, 95% confidence interval [CI] 1.116–1.878, *P* = .005) and NHR (OR = 1.480, 95% CI 1.158–1.892, *P* = .002) were identified as independent risk factors for stroke severity. Additionally, the correlation between combined NLR and NHR and AIS severity achieved a sensitivity of 81.4% and specificity of 60.4% with a best cutoff value of 6.989. This outcome was superior to that of the single composite inflammatory index. Moreover, NLR (OR = 1.252, 95% CI 1.008–1.554, *P* = .042) was an independent risk factor for poor short-term prognosis in patients with AIS. When the optimal cutoff value was 2.605, the sensitivity of NLR correlation with the short-term prognosis of AIS was 82.2%, and the specificity was 59.3%. NLR combined with NHR exhibits a strong correlation with disease severity in AIS. Meanwhile, an elevated NLR in patients with AIS can predict a poor short-term prognosis.

## 1. Introduction

A stroke occurs when focal neurological signs or symptoms caused by impaired blood flow in the brain last for at least 24 hours; 77% of strokes are first-time events.^[1]^ Globally, approximately 15 million new stroke cases occur each year, of which 5 million lead to death, while another 5 million result in permanent disability.^[2]^ The incidence of stroke has continued to increase over the past 50 years, affecting a progressively younger population, with societal lifestyle changes and increased prevalence of cardiovascular risk factors (e.g., diabetes, hypertension, dyslipidemia, and obesity).^[3]^ Acute stroke has a rapid onset and often leads to severe sequelae or even death if not treated promptly. Acute ischemic stroke (AIS) is the leading cause of acute stroke, accounting for approximately 87% of all stroke types.^[4]^ It is also the leading cause of permanent disability in adults, the second most common cause of dementia, and the third leading cause of death worldwide.^[5]^

Globally, China bears the heaviest stroke burden and the highest estimated lifetime stroke risk over 25 years.^[6]^ Meanwhile, statistical results from the 2021 Global Burden of Disease Study (1990–2019) revealed a 9.3% (95% confidence interval [CI]: 3.3–15.5%) and 3.3–15.5%) and 39.8% (95% CI, 28.6–50.7%) decrease in age-standardized morbidity and mortality, respectively, for stroke.^[7]^ However, age-standardized incidence studies in China have reported the opposite result, with a trend toward increased incidence.^[8]^ Indeed, stroke has become the leading cause of death and disability in China, with 15.7 million deaths in 2018.^[9]^

Brain injury after ischemic stroke leads to necrosis and apoptosis of brain cells, which in turn drive neuroinflammation mediated by reactive oxygen species, chemokines, and cytokines.^[10,11]^ Neuroinflammation occurs in the local and systemic microcirculation of injured brain tissue, which contributes to the progression of ischemic stroke and affects all stages of brain tissue injury and regeneration. Most nucleated cells (e.g., monocytes/macrophages, neutrophils, B cells, and T cells) can produce cytokines to regulate neuroinflammation. In particular, pro-inflammatory cytokines participate in myriad processes associated with AIS development, during which they directly contact endothelial cells, neurons, and glial cells. As such, although neuroinflammation can promote brain injury repair, excessive inflammatory cell infiltration can cause further ischemic damage to the brain, leading to larger infarct foci and more severe clinical symptoms.^[12–14]^

Recently, the neutrophil/lymphocyte ratio (NLR),^[15]^ neutrophil/high-density lipoprotein cholesterol ratio (NHR),^[16]^ platelet-to-lymphocyte ratio (PLR),^[17]^ and monocyte-to-lymphocyte ratio (MLR)^[18]^ have been identified as potential novel biomarkers of neuroinflammatory processes, which may also correlate with disease severity and prognosis in patients with ischemic stroke. Additionally, these 4 novel composite inflammatory ratios might exhibit stronger correlations with these disease variables than traditional inflammatory factors (Leukocyte; neutrophil).^[16,18,19]^ However, the relationship between these combined composite inflammation ratios and disease severity and early neurological outcome in AIS patients remains uncertain and warrants further exploration. The purpose of our study was to explore the relationship between NLR combined with NHR and disease severity and short-term prognosis in AIS patients.

## 2. Methods

### 2.1. Study population

**Figure 1.**
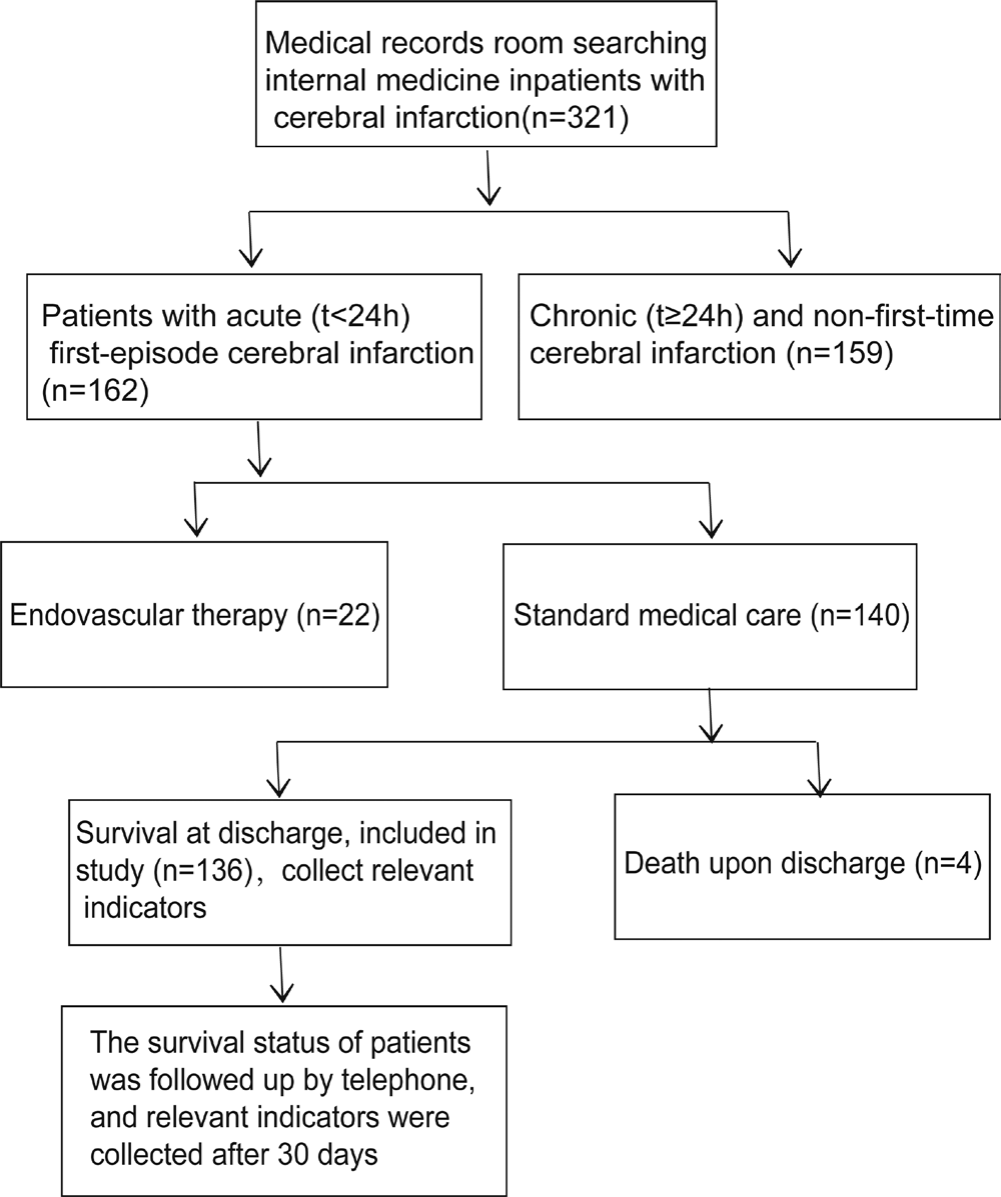
Study workflow chart. AIS = acute ischemic stroke.

A total of 136 patients with AIS who were admitted to Nantong Third People’s Hospital from September 2020 to April 2022 and received only routine medical treatment were included in this study. The workflow for this study is presented in Figure 1.

The inclusion criteria were as follows: Age ≥ 18 years; The first stroke with symptoms or signs meeting the diagnostic criteria for ischemic stroke; Diagnosis of AIS within 24 hours of onset (confirmed using non-contrast computed tomography scan and computed tomography angiography) to confirm ischemic stroke diagnosis); Head MRI performed within 48 hours; Routine blood tests completed within 24 hours of admission; Received standardized treatment for the acute phase and secondary prevention of ischemic stroke.

Exclusion criteria were as follows: Previous history of AIS or cerebral hemorrhage; Early ischemic stroke with reperfusion therapy (intravenous thrombolysis and/or mechanical embolization); Pregnancy or lactation; Severe cardiac (New York Heart Association cardiac function class III or IV or left ventricular ejection fraction < 40%), pulmonary (oxygen saturation < 90%, cyanosis, shortness of breath, abnormal blood gas analysis), liver (serum glutamate transaminase level > 10 times the upper limit of the reference interval), kidney (serum creatinine > 443 μmol/L), or neoplastic disease; Preexisting autoimmune disease; Infection at the time of diagnosis (oral temperature > 37.5 °C and white blood cell count > the upper limit of the reference interval).

This experiment was approved by the Medical Ethics Committee of Third People’s Hospital of Nantong, and the Ethics Committee reference number is EL2022024. The personal identifying information of the study population was anonymized and replaced with a coding system for privacy protection.

### 2.2. Data collection

Demographic and baseline data related to patient age, sex, history of hypertension, diabetes mellitus, hyperlipidemia, atrial fibrillation, internal carotid artery disease, and family history of stroke were obtained from medical records. The severity of ischemic stroke was assessed using the National Institutes of Health Stroke Scale (NIHSS) score on admission. Mild stroke was defined as NIHSS ≤ 5, and moderate-to-severe stroke as NIHSS score > 5. Additionally, the mRS scale was registered to define short-term prognosis at 30 days, with an unfavorable outcome being an mRS > 2.

### 2.3. Blood sampling and laboratory measurements

Blood samples were collected within 24 hours of admission, and routine laboratory testing (ions, glucose levels, and renal and liver function tests) was performed using standard methods. HDL cholesterol, monocytes, neutrophils, and lymphocytes were quantified in whole blood using an XT-1800i (Sysmex, Kobe, Japan).

### 2.4. Statistical analysis

The Kolmogorov-Smirnov test was used to assess the normal distribution of the measures. Continuous variables with a normal distribution are presented as mean ± standard deviation, while continuous variables with a non-normal distribution are presented as median (quartiles [Q25, Q75]). Independent sample *t* test and Mann–Whitney *U* test were used to analyze the differences between groups of continuous variables. The chi-square test was used to analyze categorical variables described as *n* (percentage figures) and between-group differences. The receiver operating characteristic (ROC) curve was constructed by plotting sensitivity versus 1-specificity and calculating the area under the curve (AUC). The optimal threshold was calculated based on Youden J-statistic. Furthermore, binary logistic regression models were used to test the independent effects of the NLR and NHR on AIS severity and short-term prognosis.

Due to the small sample size, we excluded similar and unrelated confounding factors. Sex, coronary heart disease, atrial fibrillation, and degree of arterial stenosis were included as confounding factors when assessing the relationship between NLR and NHR and AIS severity. Sex, age, coronary heart disease, and atrial fibrillation were applied as confounding factors when assessing the relationship between NLR and NHR and short-term prognosis. Univariate binary logistic regression analysis was carried out first, and univariate *P* < .05 was included in multivariate binary logistic regression analysis. Statistical analysis was performed using SPSS software (version 25.0; IBM Analytics) and GraphPad (prism8). *P* < .05 were considered statistically significant.

## 3. Results

### 3.1. Baseline information

A total of 136 stroke patients were included, of whom 93 (68.4%) were assigned to the mild stroke group and 43 (31.6%) were assigned to the moderate-to-severe stroke group. The coronary heart disease rate, atrial fibrillation rate, degree of carotid stenosis, white blood cell count, neutrophil percentage, apolipoprotein A1, NHR, NLR, and C-reactive protein (CRP) were higher in the moderate-to-severe stroke group than in the mild stroke group. Additionally, the proportion of male patients and ApoB/Apolipoprotein A1 were lower than those in the mild stroke group, while the remaining bioindicators did not differ significantly between the 2 groups (Table 1).

**Table 1:** Comparison of general information and clinical characteristics of patients with mild and moderately severe AIS.

Variables	Mild (93)	Moderate-to severe (43)	χ^2^/Z/t value	*P* value
Male n (%)	63 (67.7%)	21 (48.8%)	4.450	.035
Age (*X̄*±S)	70.140 ± 11.064	73.907 ± 9.990	−1.902	.059
Risk factors, n (%) High blood pressure	67 (72.0)	32 (74.4)	0.084	.772
Diabetes	41 (44.1)	18 (41.9)	0.059	.808
Coronary heart disease	5 (5.4)	10 (23.3)	9.579	.002
Atrial fibrillation	9 (9.7%)	17 (39.5%)	16.952	<.001
Degree of internal carotid artery stenosis (>50%)	19 (20.4%)	17 (39.5%)	5.514	.019
Plaque stability (unstable)	59 (63.4%)	31 (72.1%)	0.983	.321
Leukocytes×10^9^/L (*X̄*±S)	6.565 ± 1.757	8.046 ± 1.931	−4.429	<.001
Neutrophils, ×10^9^/L, M (Q1, Q3)	3.86 (3.075–4.935)	5.245 (4.478–7.575)	−5.298	<.001
Lymphocytes, ×10^9^/L(*X̄*±S)	1.744 ± 0.732	1.571 ± 0.567	1.504	.136
Mononuclear cell, ×10^9^/L (*X̄*±S)	0.517 ± 0.153	0.553 ± 0.181	−1.205	.230
Platelet count, ×10^9^/L (*X̄*±S)	200.097 ± 50.947	209.884 ± 62.460	−0.968	.335
LDH, mmol/L (*X̄*±S)	2.8188 ± 0.68929	2.912 ± 1.112	−0.595	.553
HDH, mmol/L (*X̄*±S)	1.06 ± 0.230	1.110 ± 0.200	−1.228	.222
Albumin, g/L (*X̄*±S)	39.553 ± 3.628	39.577 ± 4.050	−0.033	.974
Creatinine, μ mol/L (*X̄*±S)	69.395 ± 16.112	68.2 ± 19.407	0.376	.707
Albumin/creatinine (*X̄*±S)	0.607 ± 0.178	0.626 ± 0.1857	−0.575	.566
ApoB, g/L (*X̄*±S)	0.8318 ± 0.201	0.768 ± 0.276	1.346	.183
ApoA1, g/L (*X̄*±S)	1.217 ± 0.22	1.301 ± 0.238	−2.013	.046
ApoB/ApoA1, g/L (*X̄*±S)	0.708 ± 0.222	0.608 ± 0.242	2.366	.019
Lipoproteins, mmol/L, M (Q1, Q3)	146 (61–285)	125.5 (71–221.5)	−0.636	.525
Antitrypsin, mg/L, M (Q1, Q3)	111.9 (97.65–125.4)	109.1 (97.075–131.3)	−0.366	.714
Acidic glycoprotein, mg/L, M (Q1, Q3)	69.9 (57.6–84.45)	63.75 (59.5–83.775)	−0.503	.615
PLR, M (Q1, Q3)	119.828 (86.838–158.347)	122.215 (105.326–171.209)	−1.509	.131
NHR, M (Q1, Q3)	3.757 (2.802–4.706)	4.988 (3.935–5.881)	−3.996	<.001
MLR, M (Q1, Q3)	0.302 (0.235–0.417)	0.338 (0.252–0.437)	−1.769	.077
NLR, M (Q1, Q3)	2.341 (1.653–3.561)	3.394 (2.594–5.370)	−4.357	<.001
mRS score (at discharge, M (Q1, Q3))	1 (1–3)	4 (2.75–4)	−7.876	<.001
CRP, M (Q1, Q3)	3.6 (2.09–5.8)	6.69 (2.24–12.3)	−2.705	.007

AIS = acute ischemic stroke, ApoA1 = Apolipoprotein A1, CRP = C-reactive protein, MLR = monocyte-to-lymphocyte ratio, mRS = modified Rankin Scale, NHR = neutrophil-to- high-density lipoprotein cholesterol ratio, NLR = neutrophil-to-lymphocyte ratio, PLR = platelet-to-lymphocyte ratio.

Ninety-one (66.9%) of the 136 patients with mild stroke had a good prognosis, while the remaining 45 (33.1%) had a poor prognosis. Age, percentage of coronary artery disease, percentage of atrial fibrillation, percentage of neutrophils, NHR, NLR, PLR, MLR, and CRP levels were higher in the poor prognosis group than in the good prognosis group. Additionally, the percentage of male patients (*P* < .03) and the proportion of lymphocytes (*P* < .01) was significantly lower than in the good prognosis group (Table 2).

**Table 2:** Comparison of general information and clinical characteristics of patients with AIS with good and poor prognosis.

Projects	Good prognosis (91)	Poor prognosis (45)	χ^2^/Z/*t* value	*P* value
Male, n (%)	62 (68.1%)	22 (48.9%)	4.721	.030
Age(*X̄*±S)	69.176 ± 10.955	75.689 ± 9.283	−3.425	.001
Risk factors, n (%)				
High blood pressure	63 (69.2%)	36 (80.0%)	1.763	.184
Diabetes	41 (45.1%)	18 (40.0%)	0.313	.576
Coronary heart disease	6 (6.6%)	9 (20.0%)	5.515	.019
Atrial fibrillation	12 (13.2%)	14 (31.1%)	6.256	.012
Degree of internal carotid artery stenosis (>50%)	22 (24.2%)	14 (31.1%)	0.744	.388
Plaque stability (unstable)	60 (65.9%)	30 (66.7%)	0.007	.932
Leukocytes, ×10^9^/L (*X̄*±S)	6.854 ± 1.909	7.396 ± 1.95628	−1.533	.129
Neutrophils, ×10^9^/L, M (Q1, Q3)	3.94 (3.12–5.02)	4.85 (4.265–6.875)	−3.36	.001
Lymphocytes, ×109/L (*X̄*±S)	1.795 ± 0.723	1.474 ± 0.557	2.856	.005
Mononuclear cells, ×10^9^/L (*X̄*±S)	0.523 ± 0.154	0.539 ± 0.181	−0.554	.581
Platelet count, ×10^9^/L (*X̄*±S)	201.703 ± 52.495	206.2 ± 59.704	−0.449	.654
LDH, mmol/L (*X̄*±S)	2.805 ± 0.7015	2.935 ± 1.0782	−0.843	.401
HDH, mmol/L (*X̄*±S)	1.067 ± 0.229	1.094 ± 0.208	−0.68	.498
Albumin, g/L (*X̄*±S)	39.591 ± 3.678	39.5 ± 3.938	0.13	.897
Creatinine, μ mol/L (*X̄*±S)	69.370 ± 17.557	68.302 ± 16.491	0.34	.734
Albumin/Creatinine, M (Q1, Q3)	0.561 (0.487–0.727)	0.590 (0.500–0.723)	−0.428	.669
ApoB, g/L (*X̄*±S)	0.827 ± 0.2094	0.780 ± 0.263	1.14	.257
ApoA1, g/L (*X̄*±S)	1.221 ± 0.229	1.289 ± 0.223	−1.619	.108
ApoB/ApoA1, (*X̄*±S)	0.704 ± 0.229	0.621 ± 0.232	1.971	.051
Lipoproteins, mmol/L, M (Q1, Q3)	141.5 (60.75–269.25)	133 (70–253.5)	−0.068	.946
Antitrypsin, mg/L, M (Q1, Q3)	108.75 (96.5–125.025)	114.6 (99.05–136)	−1.191	.234
Acidic glycoprotein, mg/L, M (Q1, Q3)	65.05 (55.775–81.525)	72.7 (61.5–92.15)	−1.702	.089
PLR, M (Q1, Q3)	116.158 (86.897–144.437)	148.5294 (106.006–186.009)	−2.699	.007
NHR, M (Q1, Q3)	3.883 (2.753–4.945)	4.421 (3.701–5.582)	−2.202	.028
MLR, M (Q1, Q3)	0.299 (0.232–0.3751)	0.370 (0.286–0.460)	−2.874	.004
NLR, M (Q1, Q3)	2.259 (1.715–3.372)	3.593 (2.755–5.197)	−4.151	<.0001
CRP, M (Q1, Q3)	3.66 (2.18–5.84)	5.8 (2.215–12.65)	−1.968	.049

AIS = acute ischemic stroke, ApoA1 = Apolipoprotein A1, CRP = C-reactive protein, MLR = monocyte-to-lymphocyte ratio, mRS = modified Rankin Scale, NHR = neutrophil-to- high-density lipoprotein cholesterol ratio, NLR = neutrophil-to-lymphocyte ratio, PLR = platelet-to-lymphocyte ratio.

### 3.2. Multifactorial analysis of disease severity in patients with AIS

**Figure 2.**
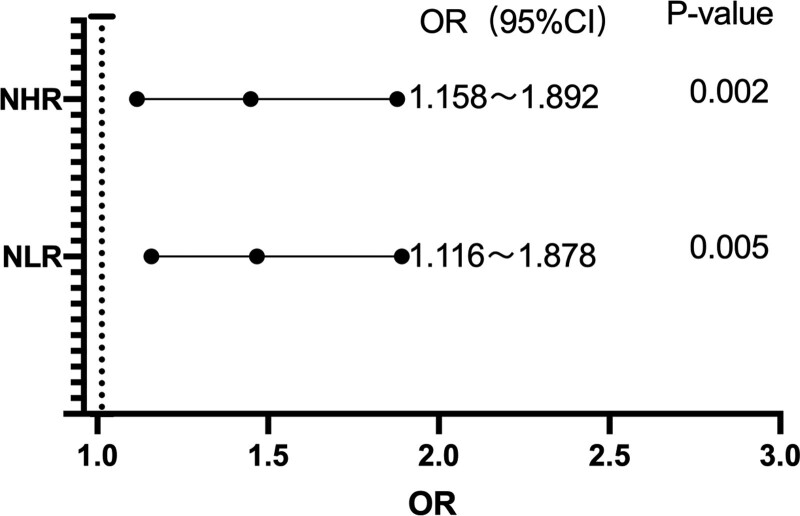
Dendrogram: correlation between NHR and NLR and disease severity. NHR = neutrophil-to-high-density lipoprotein cholesterol ratio, NLR = neutrophil-to-lymphocyte ratio.

Both NLR and NHR had significant and positive effects on AIS disease severity before and after the exclusion of confounding factors. (Table 3 and 4, Fig. 2). The crude (odds ratio [OR]: 1.449) of NLR was compared with the adjusted OR (1.472, 1.438, 1.371, 1.476, and 1.448, respectively) with sex, coronary heart disease, atrial fibrillation, and arterial stenosis excluded. Additionally, the crude odds ratio (OR: 1.467) of NLR was compared with the adjusted OR (1.462, 1.477, 1.509, 1.433, and 1.48, respectively) with sex, coronary heart disease, atrial fibrillation, and arterial stenosis excluded. Hence, sex, coronary heart disease, atrial fibrillation, and degree of arterial stenosis did not significantly interfere with the relationship between NLR and severity of AIS.

**Table 3:** Univariate Logistic regression analysis for risk factors with the severity of AIS.

	Regression coefficient	Standard Error	Wald χ2 value	*P* value	Curde OR	95% CI
NHR	0.384	0.112	11.65	.001	1.467	1.177–1.829
NLR	0.371	0.113	10.702	.001	1.449	1.075–1.669
MLR	1.895	1.079	3.083	.079	6.652	0.802–55.144
PLR	0.004	0.003	2.740	.098	1.004	0.999–1.010

AIS = acute ischemic stroke, CI = confidence interval, MLR = monocyte-to-lymphocyte ratio, NHR = neutrophil-to- high-density lipoprotein cholesterol ratio, NLR = neutrophil-to-lymphocyte ratio, OR = odds ratio, PLR = platelet-to-lymphocyte ratio.

**Table 4:** Multinomial logistic regression models for the severity of AIS.

	Regression coefficient	Standard Error	Wald χ2 value	*P* value	Curde OR	95% CI
NHR	0.392	0.125	9.827	.002	1.480	1.158–1.892
NLR	0.370	0.133	7.759	.005	1.448	1.116–1.878

AIS = acute ischemic stroke, CI = confidence interval, NHR = neutrophil-to- high-density lipoprotein cholesterol ratio, NLR = neutrophil-to-lymphocyte ratio, OR = odds ratio.

### 3.3. Multifactorial analysis of 30-d prognosis in patients with AIS

**Figure 3.**
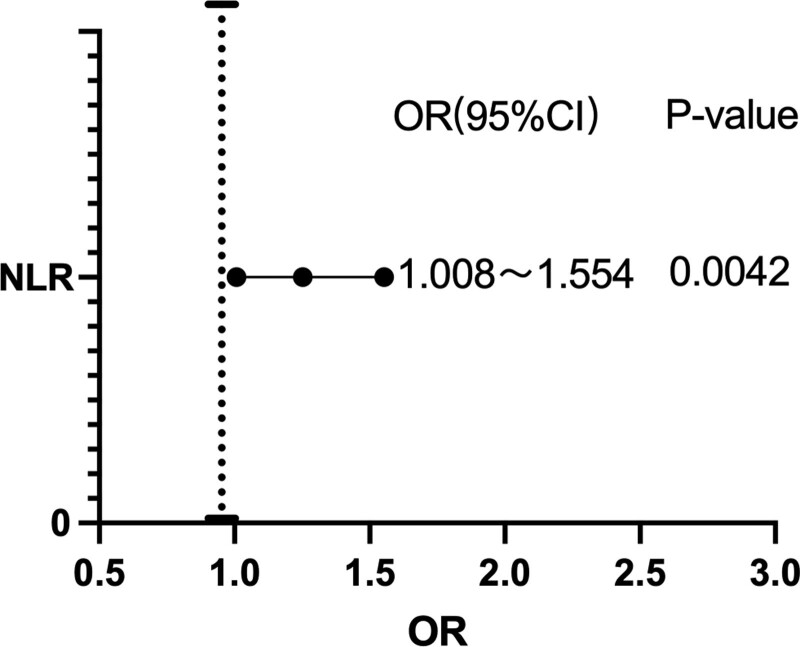
Tree diagram: correlation between NHR and NLR and prognosis. NHR = neutrophil-to-high-density lipoprotein cholesterol ratio, NLR = neutrophil-to-lymphocyte ratio.

NLR had significant and positive effects on AIS disease severity before and after the exclusion of confounding factors (Table 5 and 6, Fig. 3). The crude OR (1.330) of NLR did not change significantly when compared with the adjusted OR excluding age, sex, coronary heart disease and atrial fibrillation (1.262, 1.341, 1.315, 1.279, and 1.252, respectively). Therefore, confounding factors, such as age, sex, coronary heart disease and atrial fibrillation, did not significantly interfere with the relationship between NLR and AIS short-term prognosis.

**Table 5:** Univariate Logistic regression analysis for risk factors with 30-day prognosis of AIS.

	Regression coefficient	Standard Error	Wald χ2 value	*P* value	OR value	95% CI
NHR	0.179	0.101	3.161	.075	1.196	0.982–1.458
NLR	0.285	0.102	7.832	.005	1.330	1.089–1.623
MLR	2.652	1.141	5.402	.020	14.184	1.515–132.767
PLR	0.007	0.003	6.091	.014	1.007	1.001–1.013

AIS = acute ischemic stroke, CI = confidence interval, MLR = monocyte-to-lymphocyte ratio, NHR = neutrophil-to- high-density lipoprotein cholesterol ratio, NLR = neutrophil-to-lymphocyte ratio, OR = odds ratio, PLR = platelet-to-lymphocyte ratio.

**Table 6:** Multinomial logistic regression models for 30-day prognosis of AIS.

	Regression coefficient	Standard Error	Wald χ2 value	*P* value	OR value	95% CI
NLR	0.224	0.110	4.134	.042	1.252	1.008–1.554
MLR	1.826	1.290	2.003	.157	6.209	0.495–77.834
PLR	0.004	0.003	1.694	.193	1.004	0.998–1.01

AIS = acute ischemic stroke, CI = confidence interval, MLR = monocyte-to-lymphocyte ratio, NHR = neutrophil-to- high-density lipoprotein cholesterol ratio, NLR = neutrophil-to-lymphocyte ratio, OR = odds ratio, PLR = platelet-to-lymphocyte ratio.

### 3.4. ROC curve analysis of the relationship between NLR, NHR, and disease severity in patients with AIS

**Figure 4.**
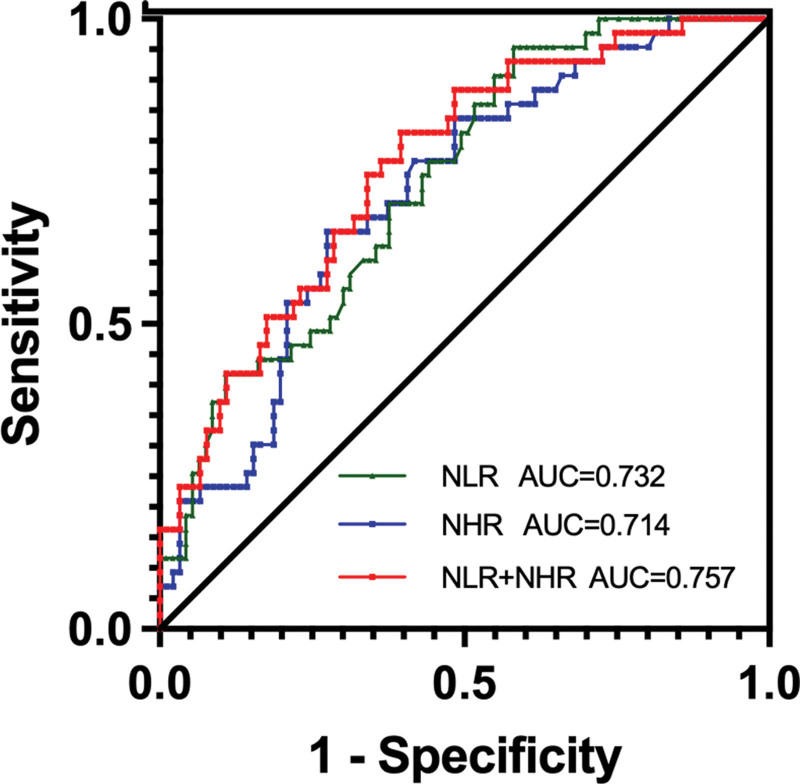
Correlation between NHR and NLR and AIS disease severity. AIS = acute ischemic stroke, NHR = neutrophil-to-high-density lipoprotein cholesterol ratio, NLR = neutrophil-to-lymphocyte ratio.

The ROC curve was plotted with the moderate-to-severe group as the state variable and NHR as the test variable. The area under the NHR curve was 0.714 (95% CI .692–.808), the sensitivity of NHR in diagnosing the severity of AIS disease was 65.1%, and the specificity was 72.5% when NHR was at the optimal cutoff value of 4.509. The ROC curve was also plotted with NLR as the test variable; the AUC was 0.732 (95% CI .647–.818), the sensitivity and specificity for NHR predicting AIS severity was 95.3% under an optimal cutoff value of 2.047. Meanwhile, the AUC of the combined NHR and NLR correlation with AIS severity was 0.756 (95% CI .732–.818 and 95% CI .732–.842), at an optimal cutoff value of 6.989, with a sensitivity of 81.4% and specificity of 60.4. (Table 7, Fig. 4).

**Table 7:** ROC curve characteristics of NHR and NLR.

Indicators	Area under the curve	Optimal cut off value	Sensitivity (%)	Specificity (%)	95% confidence interval	*P* value
NLR	0.732	2.047	95.3%	41.8%	0.647–0.818	<.001
NHR	0.714	4.509	65.1%	72.5%	0.624–0.804	<.001
NLR + NHR	0.756	6.989	81.4%	60.4%	0.732–0.842	<.001

CI = confidence interval, NHR = neutrophil-to- high-density lipoprotein cholesterol ratio, NLR = neutrophil-to-lymphocyte ratio, ROC = receiver operating characteristic.

### 3.5. ROC curve analysis of the relationship between NLR and poor prognosis in patients with AIS

**Figure 5.**
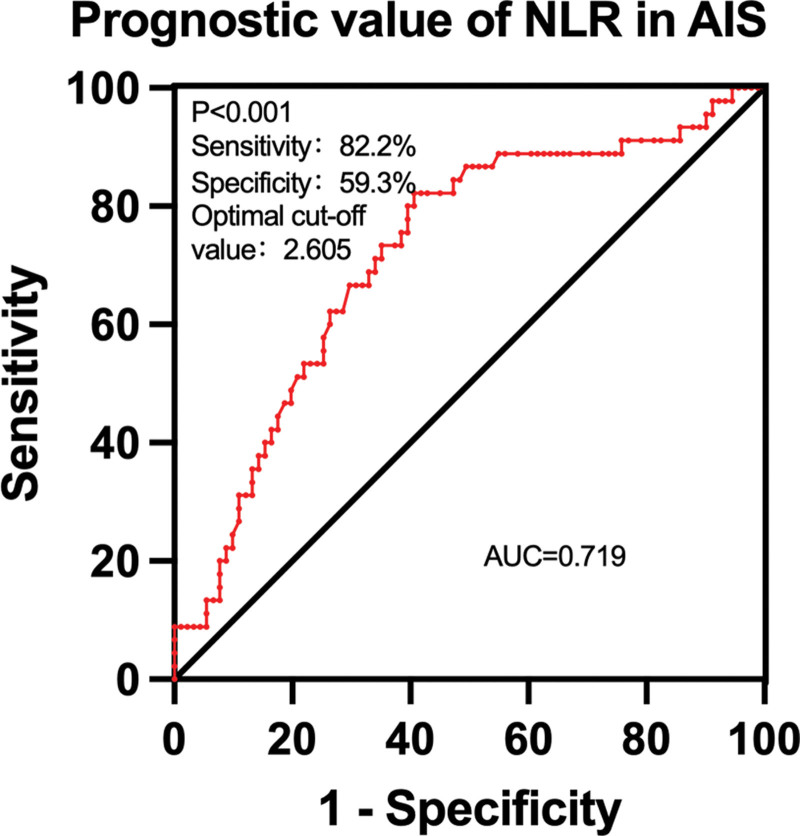
Correlation between NLR and poor prognosis in patients with AIS. AIS = acute ischemic stroke, NLR = neutrophil-to-lymphocyte ratio.

The ROC curve was constructed with a poor prognosis as the state variable and the NLR as the test variable. The area under the curve of NLR was 0.719 (95% CI: .6270–.8108); the sensitivity of NLR correlation with poor prognosis in patients with AIS was 82.2%, and the specificity was 59.3% at an optimal cutoff of 2.605 (Fig. 5).

## 4. Discussion

Interactions between various immune system cells in the acute phase of ischemic stroke are complex and regulated by multiple interconnected factors. Indeed, myriad factors impact ischemic stroke pathology, including ischemic lesion severity, the location of the stroke, age, and comorbidities. These factors affect the interactions between cell types in the necrotic brain parenchyma as well as cell-to-cell homeostasis.^[12]^ In the current study, stroke comorbidities (coronary artery disease, atrial fibrillation) and older age exacerbate disease progression in ischemic stroke, leading to poor prognosis. Moreover, consistent with the findings of the American Heart Association and American Stroke Association,^[1,12]^ stroke was strongly correlated with patient sex (male/female = 1.6:1); however, disease severity and short-term prognosis were better in male patients than in females.

The initial phase of ischemic brain injury upregulates chemokine production by leukocytes and associated adhesion molecules in endothelial cells.^[20–22]^ That is, blood leukocytes at the site of brain injury adhere to endothelial cells by adhesion molecules, which are then activated by chemokines to release pro-inflammatory cytokines, producing an inflammatory cascade response. Neutrophils are the first peripheral immune cells to infiltrate injured tissues within hours of a stroke and persist for up to 1 week after the initial ischemic injury. Neutrophils can either mechanically block microcirculation in the brain or secrete vasoconstrictors, cytokines, reactive oxygen species, and enzymes with hydrolytic activity. Additionally, neutrophils produce matrix metalloproteinase-9, which disrupts the blood-brain barrier, enhances brain edema, leads to hemorrhagic transformation in acute ischemic stroke, and aggravates brain injury, leading to poor prognosis.^[21]^ Following neutrophil invasion, monocytes adhere to the vessel wall.^[14,23]^

In contrast to neutrophils, the number of lymphocytes decreases after cerebral ischemia, thus, increasing the NLR.^[13]^ Meanwhile, high-density lipoprotein cholesterol (HDL-C) can regulate macrophages and adipocytes via cholesterol transporters, exhibiting anti-inflammatory effects.^[24]^ Additionally, HDL-C maintains endothelial function and low blood viscosity and exhibits antiatherogenic properties. Therefore, high HDL-C levels reduce the risk of poor prognosis in patients with atherosclerotic stroke.^[16]^ In contrast, the increased proportion of neutrophils among inflammatory cells leads to more severe AIS and worse prognosis; the opposite effect is observed for lymphocytes.

Numerous studies have confirmed that NLR and NHR are reproducible composite biomarkers that can predict the severity of the systemic inflammatory load in ischemic stroke.^[25]^ Moreover, NLR is associated with early neurological deterioration in acute stroke patients^[19]^ and can predict the risk of hemorrhagic transformation in AIS patients as a biomarker for risk-stratified patients and poor prognosis in AIS.^[26,27]^ In fact, Wei et al reported that NLR might have a greater negative diagnostic value in predicting stroke-associated pneumonia in patients with AIS.^[28]^ Meanwhile, Zhang et al^[29]^ found NHR to be a reliable and simple independent predictor of hemorrhagic transformation in patients with AIS. Chen et al^[16]^ further revealed that high NHR levels are associated with poor prognosis 3 months after intravenous thrombolysis in patients with AIS.

Although many studies have assessed the prognostic effects of NLR and NHR as individual indicators of AIS, no previous studies have evaluated the predictive value of combined NLR and NHR on AIS disease severity and prognosis. As such, the current study provides novel insights regarding the correlation between composite inflammatory indexes and AIS. Ultimately, these findings can facilitate early prediction of AIS disease prognosis and severity, thus improving clinical outcomes.

The combined use of NLR, NHR, HDL-C, and inflammatory markers (neutrophils and monocytes) to diagnose AIS severity and prognosis has 2 advantages. First, the combined use of NLR and NHR comprehensively evaluates pro-inflammatory factors, lipids, and atherosclerosis in patients with AIS; this combined index is more stable than single blood parameters that may be influenced by multiple variables, including dehydration, hyperhydration, and blood specimen handling procedures.^[30]^ Moreover, the composite NHR and NLR index exhibited stronger correlations with disease severity and short-term prognosis than MLR or PLR. The composite inflammatory ratio (NLR and NHR) was also more strongly correlated with AIS severity and prognosis (sensitivity, 81.4%; specificity, 60.4%) than either individual indicator. Meanwhile, NLR was associated with poor short-term prognosis in AIS, while NHR was not an independent risk factor for this variable.

Several limitations were noted in this study. First, it is a retrospective study, and there is data loss during the study. Second, our data for the composite inflammatory ratios MLR, PLR, NLR, and NHR were collected upon admission. However, the results show that these biomarkers may fluctuate at different times in AIS. Third, this study focused exclusively on patients who had suffered their first AIS within 24 hours of observation. As such, the results are not directly applicable to other types of strokes. Fourth, although we attempted to adjust for all potential influencing factors, etiology, disease severity, and treatment may impact poststroke outcomes more than inflammatory indices. Finally, neurological recovery also depends largely on the standard of care and rehabilitation compliance after a stroke. Patient compliance may also influence the prognostic value of various parameters for long-term outcomes in ischemic stroke.

## 5. Conclusion

This retrospective study shows that lower NLR and NHR levels upon admission in patients with AIS may be valid indicators of less severe clinicopathological features. Hence, NLR combined with NHR might provide a more comprehensive assessment of clinicopathological features in AIS. Furthermore, higher levels of NLR upon admission might predict a worse short-term prognosis for patients with AIS.

## Acknowledgments

We would like to thank the doctors in the Department of Neurology of the Third People’s Hospital of Nantong City for assisting us in diagnosing diseases and collecting data.

## Author contributions

**Conceptualization:** Jian-Hua Song, Guo-Xiang Huang.

**Data curation:** Feng Zhu, Yan Ji, Yun-Feng Zhang.

**Formal analysis:** Feng Zhu, Jian-Hua Song.

**Funding acquisition:** Yan Ji.

**Investigation:** Guo-Xiang Huang.

**Software:** Guo-Xiang Huang.

**Supervision:** Yun-feng Zhang.

**Writing – original draft:** Feng Zhu.

**Writing – review & editing:** Yun-Feng Zhang.
